# A Rare Case of Cutaneous Lymphomatoid Granulomatosis: A Case Report and Literature Review

**DOI:** 10.7759/cureus.108448

**Published:** 2026-05-07

**Authors:** Jerel A Phillips, Ayushi Awale, Comfort Anim-Koranteng, Marie Thearle, Virgil Hatcher

**Affiliations:** 1 Internal Medicine, Columbia University College of Physicians and Surgeons, Harlem Hospital Center, New York City, USA; 2 Rheumatology, University of Cincinnati College of Medicine, Cincinnati, USA; 3 Internal Medicine, Harlem Hospital Center, New York City, USA; 4 Dermatology, Columbia University College of Physicians and Surgeons, Harlem Hospital Center, New York City, USA

**Keywords:** cutaneous, dermatology, lymphomatoid granulomatosis, oncology, rare, skin biopsy, ulcer

## Abstract

Lymphomatoid granulomatosis is a rare lymphoproliferative disease caused by dysfunction of host surveillance of Epstein-Barr virus (EBV)-infected B cells, which predominantly affects male adults, with a median onset at 46 years. Diagnosis can be challenging due to its nonspecific symptoms and the need for careful histopathological evaluation, particularly in atypical presentations. Here, we present a case of a 42-year-old Black man from Senegal with no past medical history who presented with a chronic, non-healing ulcer on his right upper extremity that had worsened over six months. Family history revealed a sister with kidney disease who required early dialysis. Initial vital signs showed a blood pressure of 178/123 mmHg and a heart rate of 127 beats/min, with normal temperature and oxygen saturation. A 4 × 5 cm crusted, non-tender stage 3 ulcer with hyperpigmented edges was found on his right arm. Laboratory tests showed pancytopenia, stage 5 chronic kidney disease with nephrotic-range proteinuria, and normocytic anemia. Blood cultures, HIV, and leishmaniasis tests were negative, and chest radiograph findings were normal. Initial evaluation also revealed chronic hepatitis B infection. CT raised concern for osteomyelitis, but MRI was negative. Skin biopsy showed nonspecific inflammatory changes, and cultures revealed methicillin-sensitive *Staphylococcus aureus*. He received antibiotics with minimal improvement. Five weeks later, he was readmitted with worsening constitutional symptoms, uremia requiring dialysis, and progression of the skin lesion. A repeat skin biopsy revealed an EBV-positive lymphoproliferative disorder consistent with grade 3 lymphomatoid granulomatosis, with predominance of CD4+ T cells. Molecular studies revealed an ATM gene mutation (p.Asn2282Ser) and acquired trisomy X with a normal Y chromosome. The ulcer improved without any further intervention. This case underscores the need for heightened clinical suspicion, repeat tissue sampling, and early dermatologic evaluation, which are critical for diagnosis.

## Introduction

Lymphomatoid granulomatosis (LyG) is a rare angiocentric and angiodestructive Epstein-Barr virus (EBV)-linked B-cell lymphoproliferative disorder in a T-cell-rich background [1]. While presentation predominantly involves the lungs [1], we present a rare case of cutaneous-predominant LyG without lung involvement, which required multiple skin biopsies to establish the diagnosis.

This article was previously presented as a poster at the 2025 New York Health + Hospitals/Harlem Residents & Fellows’ Research Fair on April 10, 2025.

## Case presentation

**Figure 1:**
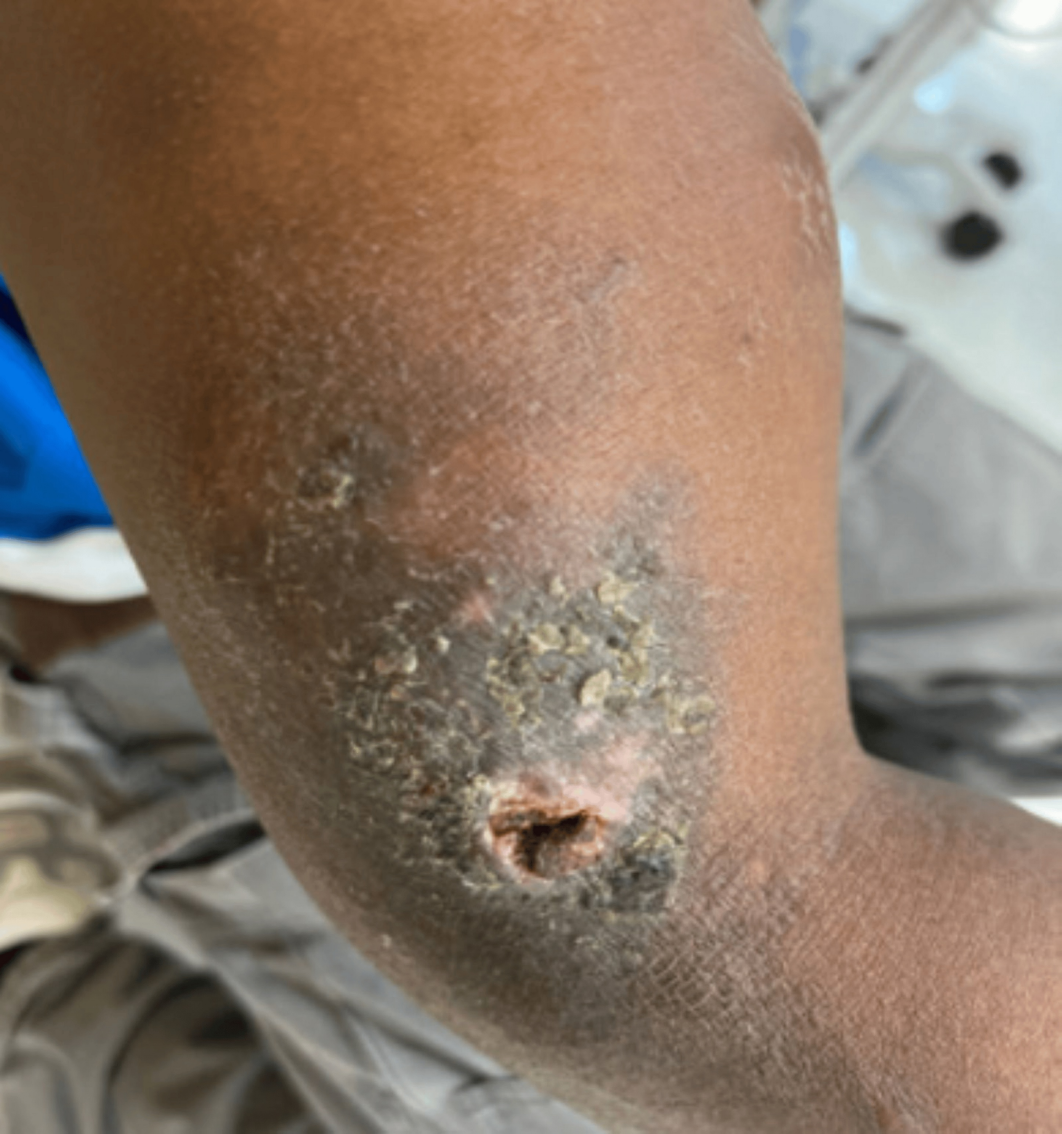
Initial presentation of the ulcer of the right upper extremity showing an indurated swelling with a chronic, crusted, non-tender skin ulcer measuring 4 x 5 cm with a depth down to the subcutaneous fascia and a hyperpigmented edge on the lateral aspect of his right upper extremity near the elbow.

A 42-year-old previously healthy Black Senegalese man, living in the USA for one year, presented to the Emergency Department (ED) with a six-month history of a non-healing ulcer on his right upper extremity (RUE), which initially began as a small papule, with associated swelling and pain. Family history was notable for kidney disease in a sister who required dialysis at an early age. Vital signs revealed elevated blood pressure (178/123 mmHg) and tachycardia (127 bpm), with normal temperature (37.6°C) and oxygen saturation (100%). Examination revealed a 4 × 5 cm ulcer on his right upper extremity with induration, hyperpigmented borders, and extension to the subcutaneous fascia, but no crepitus (Figure 1). 

Initial labs revealed normocytic anemia (hemoglobin 9.9 g/dL, mean corpuscular volume (MCV) 86 fL), leukopenia (WBC 3.71 × 10⁹/L), elevated creatinine (5.4 mg/dL), and nephrotic-range proteinuria (>5.2 protein/creatinine ratio). Chest radiograph was unremarkable. Computed tomography without contrast of the RUE revealed subcutaneous edema and fat stranding along the lateral aspect of the distal humerus with a questionable periosteal reaction, raising concern for early osteomyelitis, though MRI later ruled this out. Renal ultrasound showed medical renal disease and incidental splenomegaly. Wound cultures grew methicillin-sensitive Staphylococcus aureus (MSSA). Blood cultures, HIV, tuberculosis, and leishmaniasis testing were negative, but chronic inactive hepatitis B was identified with a positive PCR and reactive antigen and core antibody.

Four punch biopsies from the ulcer margin performed on hospital day 3 revealed papillated squamous epithelial hyperplasia with dermal fibroplasia and mixed inflammatory infiltration. He was discharged after six days of cefpodoxime and clindamycin, followed by seven days of cephalexin.

**Figure 2:**
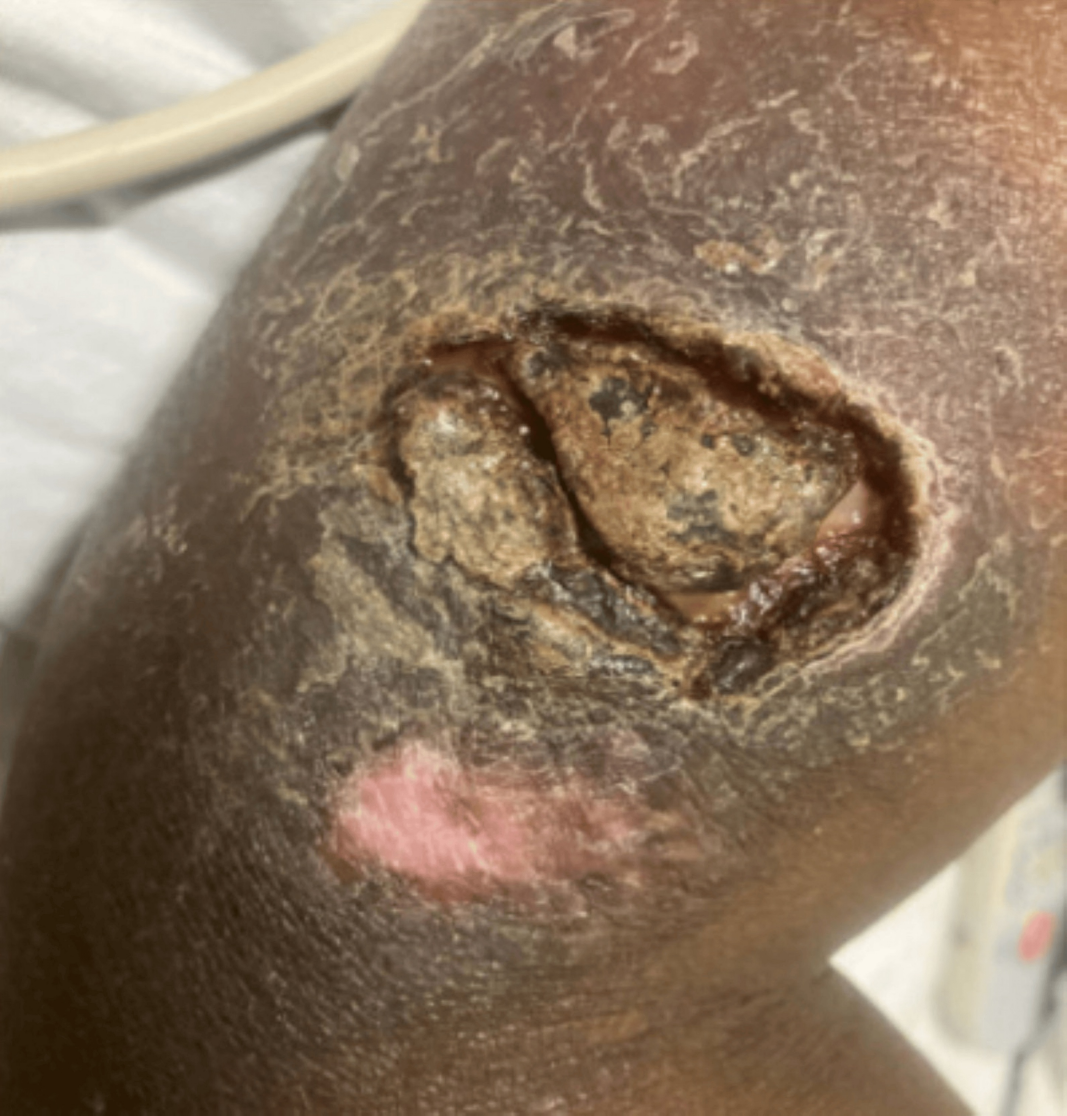
Worsening, non-healing right upper extremity ulcer upon readmission with enlargement.

Five weeks later, he was readmitted with a three-day history of fever, night sweats, vomiting, diarrhea, and worsening RUE ulceration (Figure 2). 

Vital signs were notable for elevated blood pressure (171/101 mmHg) and a temperature of 37.2 °C. Labs revealed end-stage renal disease (ESRD) (creatinine 13 mg/dL) and leukopenia. Empiric antibiotics (cefepime and vancomycin) were initiated, and hemodialysis was begun. Tissue culture grew Staphylococcus haemolyticus, and antibiotics were switched to cephalexin, which was given for six days. Blood cultures were negative.

**Figure 3:**
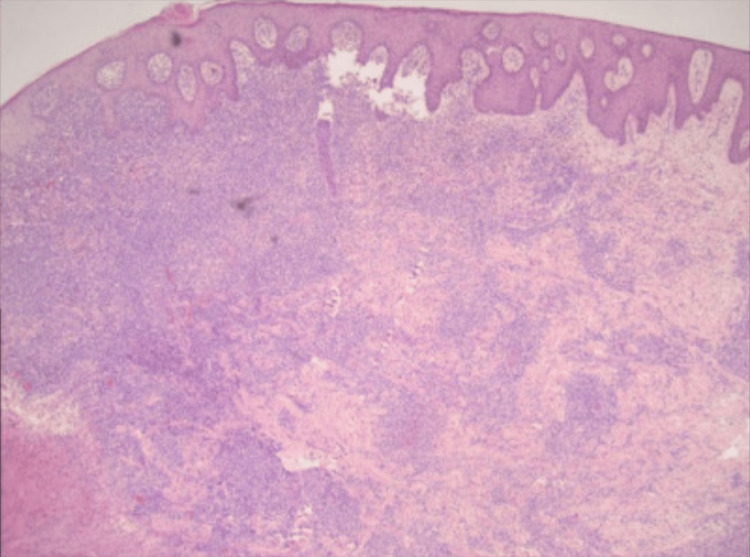
Photomicrograph of skin biopsy specimen showing an angiocentric and angiodestructive, polymorphous lymphoid infiltrate in the dermis with variable numbers of histiocytes and plasma cells and >50 large EBV positive B-cells per high power field, consistent with Grade 3 LyG.

A repeat punch biopsy of the RUE ulcer edge performed on hospital day 11 revealed >50 EBV-positive cells per high-power field, consistent with grade 3 LyG (Figure 3), with subsequent positive EBV PCR serology (274 IU/L). 

Histopathology demonstrated CD20+ atypical B cells and a predominance of small reactive CD4+ versus CD8+ T cells. No high-level tumor mutation burden or microsatellite instability was identified, but genetic testing revealed a rare ataxia-telangiectasia mutated (ATM) gene mutation (p.Asn2282Ser), not reported as a somatic variant in tumors (COSMIC), but as a rare population variant in publicly available databases (gnomAD). Trisomy X with an acquired large copy gain of an X chromosome and a normal Y chromosome was also identified.

**Figure 4:**
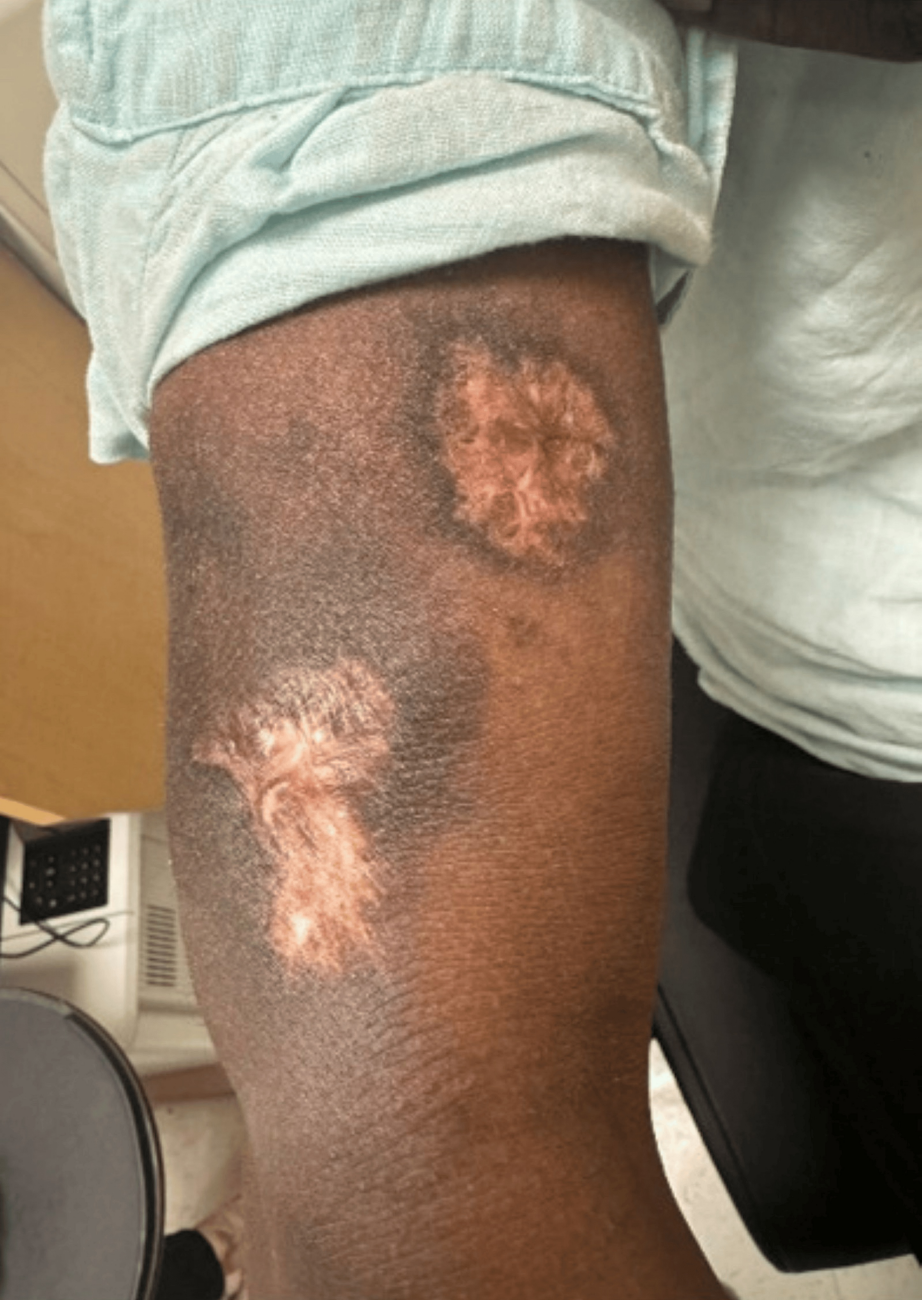
Appearance of the patient’s healed right upper extremity ulcer with depigmented scarring, 85 days after the initial presentation.

The patient was discharged on hospital day 29 with oncology and dermatology follow-up. The WBC count was 5.24 K/uL, and the ulcers were healed with depigmented scarring (Figure 4) at follow-up. 

## Discussion

Our patient’s atypical presentation and course, lack of lung involvement, and second skin biopsy results highlight the crucial role of re-biopsy in unclear cases to establish a diagnosis of LyG.

LyG, a rare disease with unknown prevalence first described in 1972, usually has pulmonary involvement [1], shows a middle-aged male predominance [2], and has a median survival of 14 months from diagnosis [3], with mortality due to respiratory failure [4]. Within the Incidence-SEER Limited Field Database November 2024 update [2], eight, three, and three sporadic cases were reported, respectively, in 2022, 2021, and prior to 2021, with at least half of cases being male, 75% White (none Black), and more than 80% over 50 years old. Two-thirds of cases presented in the lung or intrathoracic lymph nodes, and only one presented in cutaneous tissue.

Skin lesions, with or without ulceration and ranging from papules to plaque lesions of any color [3], are reportedly seen in one-fifth of cases and can be the initial manifestation, sometimes preceding lung involvement by years, with isolated presentation possibly representing a benign end of the spectrum [5]. Differential diagnoses include vasculitis, erythema nodosum, infection, mycobacteriosis, granuloma annulare, sarcoidosis, and lymphoma.

LyG is histologically defined by angiocentric, angiodestructive infiltrates composed of EBV-infected B cells and reactive T cells and is classified into three grades based on the number of EBV-positive B cells. Grade 3 LyG is often monoclonal and represents high-grade disease and is hypothesized to be immune-independent. Progression to overt T-cell lymphoma can occur in up to 20% of patients [6]. Despite his initial presentation with leukopenia and systemic involvement, our patient fared better than expected, possibly because he lacked poor prognostic factors such as persistent pancytopenia, neurologic manifestations, hepatomegaly, and large numbers of atypical lymphoreticular cells on biopsy [7].

CD4-positive T cells usually predominate [1], while ATM is essential in cell cycle regulation and can be involved in EBV-related malignancies due to impaired viral genome control [8]. Although there is no direct link between ATM mutations and trisomy X and the subsequent development of LyG, the ATM mutation could have contributed to the acquisition of additional X chromosomes, a potential poor prognostic sign, increasing susceptibility to develop grade 3 LyG. With a possible deficiency of CD8-positive cytotoxic cells, along with the ATM mutation and trisomy X, immune dysregulation, lymphomagenesis, and the severity of his cutaneous lesion may have been the result. Therefore, further studies into the association of the ATM gene mutation and trisomy X with LyG should be conducted to establish a connection.

Grading of LyG is important for treatment, as historical outcomes have been poor, with a median survival of 14 months [1]. Clinical trials with interferon-α2b have shown some success in low-grade LyG [3]. There has been increased progression-free survival (PFS) to 56% (median follow-up 5.1 years) in patients with grades 1 and 2 LyG, and to 44% (median follow-up 32 months) in those with grade 3 LyG treated with immunochemotherapy (dose-adjusted etoposide, prednisone, vincristine, cyclophosphamide, doxorubicin, rituximab, DA-EPOCHR) [1]. Therefore, early diagnosis is crucial.

## Conclusions

We report a rare case and atypical presentation of high-grade LyG, which manifested solely with cutaneous involvement. This presentation is seen in fewer than one quarter of cases and can precede systemic involvement by months or years, mimicking infection, vasculitis, or autoimmune disease. EBV-positive grade 3 LyG carries a high risk of systemic progression. The presence of an ATM mutation and trisomy X may reflect underlying genetic instability contributing to its pathogenesis, but this cannot be definitively concluded. Therefore, further research into the genetic factors, clinical behaviors, and associations of LyG is warranted. Early dermatological review is crucial to diagnosis and treatment strategies, especially when the clinical presentation is atypical.
